# Association of polymorphisms in the 5′ untranslated region of *RAD51* gene with risk of endometrial cancer in the Polish population

**DOI:** 10.1007/s00404-014-3305-6

**Published:** 2014-06-15

**Authors:** Magdalena M. Michalska, Dariusz Samulak, Hanna Romanowicz, Beata Smolarz

**Affiliations:** 1 Department of Obstetrics and Gynaecology, Regional Hospital in Kalisz, Kalisz, Poland; 2 Cathedral of Mother’s and Child’s Health, Poznan University of Medical Sciences, Poznan, Poland; 3 Clinic of Gynaecological Surgery, Poznan University of Medical Sciences, Poznan, Poland; 4 University of Computer Sciences and Skills, Rzgowska 17, Lodz, Poland; 5 Department of Fetal-Maternal Medicine and Gynecology, Institute of Polish Mother’s Memorial Hospital, Rzgowska 281/289, 93-338 Lodz, Poland

**Keywords:** Endometrial cancer, RAD51, Gene polymorphism

## Abstract

**Purpose:**

Many of the studies have analyzed cell repair capabilities, following cancer development. The cellular reaction to DNA damaging agents can modulate the susceptibility to various tumors. This reaction is mainly determined by DNA repair efficacy which, in turn, may be influenced by the variability of DNA repair genes, expressed by their polymorphisms.

**Methods:**

This report describes studies of the distribution of genotypes and the frequency of alleles of the G135C (rs1801320) and G172T (rs1801321) *RAD51* polymorphism in 630 paraffin-embedded samples of tumor tissue from patients with endometrial cancer. DNA from 630 normal endometrial tissues served as control. *RAD51* polymorphisms were determined by PCR–RFLP.

**Results:**

In the present work, a relationship was identified between *RAD51* G135C polymorphism and the incidence of endometrial cancer. Endometrial cancer patients had an overrepresentation of 135C allele. The 135C/C homozygous variant increased cancer risk. A tendency towards a decreased risk of endometrial cancer was observed with the occurrence of combined G135C–G172G genotype of *RAD51* polymorphism. An association was confirmed between *RAD51* G135C and G172T polymorphisms and endometrial cancer progression, assessed by the histological grades.

**Conclusions:**

The results support the hypothesis that *RAD51* G135C and G172T polymorphisms may be associated with endometrial cancer occurrence and/or progression.

## Introduction

Endometrial cancer (EC) is the fourth among all malignant neoplasms in women, after breast cancer, lung cancer and large intestine cancer [1–3].

Prognostic factors (age, hormonal condition, diabetes, hypertension, obesity, sterility, low birth number, late menopause and genetic factors) commonly used for the identification of EC present an incomplete picture of the tumor biology of endometrial cancer [1]. Therefore, investigation of other prognostic factors is of special clinical relevance, particularly in view of the unexpectedly progressive course of the disease and frequent relapses in some cases.

Double-strand DNA breaks (DSBs) are the most dangerous DNA damage [4]. If not repaired, they cause loss of chromosomes and cell death [4]. An accumulation of DSBs destabilizes the genome and rearranges it, leading to down-regulation of transcription and development of various cancer diseases [4].

Double-strand DNA breaks are repaired by the following two mechanisms: recombination (HR) and non-homologous end joining (NHEJ) [5].

RAD51 homolog (RecA homolog, *Escherichia coli*) (*Saccharomyces cerevisiae*) is involved in the homologous recombination and repair of double-strand breaks in DNA and DNA cross-links, as well as in the maintenance of chromosome stability [4].

Literature data suggest that RAD51 levels do not generally increase in normal cells [6]. Raderschall et al. [7] showed that increased levels of RAD51 in tumor cells were found to be associated with unscheduled HR and genetic instability. Therefore, we suggest that the elevated levels of RAD51 may be signalling the presence of extensive DNA damage.

Changes in RAD51 biosynthesis are usually preceded by changes in its gene transcription and mRNA level [8]. Gene variability could contribute to the level of RAD51 biosynthesis.

*RAD51* gene is highly polymorphic: two common *RAD51* single nucleotide polymorphisms (SNPs) G135C (rs1801320) and G172T (rs1801321) in the 5′UTR have been reported to be associated with altered gene transcription [9, 10]. It is suggested that these two polymorphisms, located at the regulatory locus of *RAD51* promoter, are associated with mRNA expression [10].

Polymorphisms in DNA repair genes, such as *RAD51,* may alter the activity of the proteins and, thus, modulate cancer susceptibility [11–17].

A large number of molecular epidemiologic studies have been performed on various neoplasms, such as cancer of breast, bladder, lung, head and neck and skin to evaluate the role of *RAD51* polymorphisms [18–22].

In the literature, many reports confirm that *RAD51* G135C and G172T polymorphisms may be associated with the development of certain types of cancers [14, 15, 23–27], but little is known about their association with endometrial carcinoma.

The study of *RAD51* G135C polymorphism in Polish population identified a haplotype associated with endometrial cancer [28–30]. Romanowicz et al. [28] showed that the *RAD51* C135C genotype is associated with the risk of EC in Polish women. Similar results were obtained by Krupa et al. [30] in Polish population. Smolarz et al. [29] found the correlation between the *RAD51* G135C polymorphism and endometrial cancer. The *RAD51* 135C allele was associated with a significantly increased risk of endometrial cancer in Poland [30].

A recent study on the Polish populations has provided the first epidemiological evidence supporting a connection between *RAD51* gene variants and the development of endometrial premalignant lesions [28–30].

These data prompted us to search for an association between EC occurrence and the G135C (rs1801320) and G172T (rs1801321) single nucleotide polymorphisms in *RAD51* gene.

## Materials and methods

### Patients

A total of 630 patients with histologically proven diagnosis of endometrial cancer were included in the reported study. The full characteristics of the study group are presented in Table 1. Paraffin embedded tumor tissue specimens were obtained from postmenopausal women with endometrial carcinoma, treated at the Department of Menopausal Diseases, Institute of Polish Mother’s Memorial Hospital (Lodz, Poland), between 1998 and 2012. All the diagnosed tumors were graded by criteria of the International Federation of Gynaecology and Obstetrics (FIGO) [31]. Control samples consisted of DNA extracted from normal endometrial tissue from age-matched 630 cancer-free women. Normal endometrial specimens were obtained from patients who had undergone hysterectomy for intramural leiomyomas.

**Table 1:** Characteristics of the study populations

Characteristics	Number of cases (%)
Age (years)	
Median	69
Range	50–84
BMI (body mass index) (kg/m^2^)	
<24.9	120 (19 %)
25–29.9	222 (35 %)
>30	288 (46 %)
Number of pregnancies	
1	222 (36 %)
2–3	408 (64 %)
>4	0
Use of hormone replacement therapy, HRT	
Yes	432 (69 %)
No	198 (31 %)
First menarche	
Before 11 years	80 (12 %)
12–13 years	320 (51 %)
14–15 years	170 (27 %)
After 16 years	60 (10 %)
FIGO grade	
G1	180 (29 %)
G2	420 (67 %)
G3	30 (5 %)
FIGO stage	
I	174 (28 %)
II	441 (70 %)
III	15 (2 %)
Menopause status	
Postmenopausal	630
Uterine bleeding	
Yes	450 (71 %)
No	180 (29 %)
Endometrial transvaginal ultrasound, TVU	
>5 mm	543 (86 %)
Diabetes mellitus	
Yes	186 (30 %)
No	444 (70 %)
Hypertension	
Yes	360 (57 %)
No	270 (43 %)

*n* = 630

### DNA isolation

Endometrial tissue samples (cancerous and non-cancerous) were routinely fixed in formaldehyde, embedded in paraffin, cut into thin slices and stained with haematoxylin/eosin for pathological examination. DNA for analysis was obtained from archival pathological paraffin-embedded (Department of Pathology, Institute of the Polish Mother’s Memorial Hospital, Lodz, Poland) samples, which were deparaffinized in xylene and rehydrated in ethanol and distilled water. In order to ensure that the chosen histological material was representative for cancerous and non-cancerous tissue, each sample qualified for DNA extraction was initially checked by a pathologist. DNA was extracted from the material, using a commercially available QIAmp Kit (Qiagen, Hilden, Germany), according to the manufacturer’s instruction. The Local Ethics Committee approved the study and each patient gave a written consent (No 4/2011).

### Genotyping

Single nucleotide polymorphism G135C of *RAD51* gene was determined by polymerase chain reaction–restriction fragment length polymorphism (PCR–RFLP), using the following primers: 5′-TGG GAA CTG CAA CTC ATC TGG-3′ (forward) and 5′-GCG CTC CTC TCT CCA GCA G-3′ (reverse) [20].

The PCR analysis was carried out in a PTC-100TM (MJ Research, INC, Waltham, MA, USA) thermal cycler. All PCRs were carried out in a volume of 25 μl, containing 100 ng of genomic DNA, 0.2 μmol of each appropriate primer (ARK Scientific GmbH Biosystems, Darmstadt, Germany), 2.5 mM MgCl_2_, 1 mM dNTPs and 1 U of Taq Polymerase (Qiagen GmbH, Hilden, Germany).

*RAD51* G135C genotyping was analyzed by PCR amplification of a 175-bp region around nucleotide 135. That region contained a single *Mva*I site that was abolished in the 135C allele. Wild-type alleles were digested by *Mva*I (New England BioLabs, Frankfurt am Main, Germany), resulting in 86- and 71-bp products. The 135C allele was not digested by the enzyme, resulting in a single 157-bp product.

The PCR cycle conditions included 94 °C for 60 s, 54 °C for 30 s then 72 °C for 40 s, repeated for 35 cycles. Following the digestion with *Mva*I for 4 h at 37 °C, the samples were run on 2 % agarose gel and visualized by ethidium bromide staining. Each subject was classified into one of the three possible genotypes: 135G/G, 135G/C or 135C/C.

Single nucleotide polymorphism G172T of *RAD51* gene was analyzed by the PCR–RFLP technique, using the following primers: 5′-TGG GAA CTG CAA CTC ATC TGG-3′ (forward) and 5′-GCT CCG ACT TCA CCC CGC CGG-3′ (reverse) [21].

The PCR profile for G172T polymorphism consisted of an initial melting step at 95 °C for 5 min, followed by 30 cycles of 95 °C for 30 s, 65 °C for 45 s and 72 °C for 50 s plus a final extension step of 72 °C for 10 min. The product after PCR was digested with *NgoM*IV (New England BioLabs, Frankfurt am Main, Germany) overnight. The products were separated in 2 % agarose gel. The 172G/G genotype produced two bands (110 and 21 bp), whereas the 172T/T genotype produced only one band (131 bp) and the 172G/T heterozygote displayed all the three bands (131, 110 and 21 bp).

### Statistical analysis

The observed numbers of each *RAD51* genotype were compared with those expected for a population in Hardy–Weinberg equilibrium (HWE), using the Chi-square test. The genotypic-specific risks were estimated as odds ratios (ORs) with associated 95 % intervals (CIs) by unconditional logistic regression. The wild-type alleles were used as reference groups. OR for each combination was calculated with homozygous wild-type variant combination as reference. *p* values <0.05 were considered significant. All the statistical analyses were performed using the STATISTICA 6.0 software (Statsoft, Tulsa, Oklahoma, USA).

## Results

All the recruited samples were successfully genotyped for *RAD51* polymorphisms. After the PCR analysis, all the patients and controls were classified into three genotypes of the G135C polymorphism, namely 135G/G, 135G/C and 135C/C, and of the G172T polymorphism, namely 172G/G, 172G/T and 172T/T.

The genotype frequency of the *RAD51* G135C and of the *RAD51* G172T single nucleotide polymorphisms in the endometrial cancer samples and controls has been summarized in Table 2.

**Table 2:** The allele and genotype frequency and odds ratio (OR) of G135C polymorphism of the *RAD51* gene in endometrial cancer and controls

*RAD51* G135C	Patients (*n* = 630)		Controls (*n* = 630)		OR (95 % CI)^a^	*p* ^b^
	Number	(%)	Number	(%)		
G/G	129	20	189	30	1.00 Ref	
G/C	135	22	297	47	**0.66 (0.49–0.90)**	**0.010**
C/C	366	58	144	23	**3.72 (2.77–5.00)**	**<0.0001**
G	393	31	675	54	1.00 Ref	
C	867	69	585	46	**2.54 (2.16–2.99)**	**<0.0001**

Data in boldface are statistically significant

^a^Crude odds ratio (OR); 95 % CI, confidence interval at 95 %

^b^Chi-square

It can be seen from this Table 2 that there are significant differences in the frequency of *RAD51* G135C genotypes (*p* < 0.05) between the two investigated groups. A weak association was observed between endometrial carcinoma occurrence and the presence of 135C/C and 135G/C genotypes. A stronger association was observed for 135C/C than for 135G/C heterozygous variant. Variant C allele of *RAD51* increased cancer risk. That increase was statistically significant (*p* < 0.05). In case of the G135C polymorphism of *RAD51* gene the distribution of the genotypes in the patients differed significantly from one expected from the Hardy–Weinberg equilibrium (*p* < 0.05).

No statistically significant differences were observed in genotype frequencies of *RAD51* G172T polymorphism between the control group and the EC patients (see Table 3). Among the patients, all genotype distributions did not differ significantly (*p* > 0.05) from those expected by the HWE.

**Table 3:** The allele and genotype frequency and odds ratio (OR) of G172T polymorphism of the *RAD51* gene in endometrial cancer and controls

*RAD51* G172T	Patients (*n* = 630)		Controls (*n* = 630)		OR (95 % CI)^a^	*p* ^b^
	Number	(%)	Number	(%)		
G/G	159	25	177	28	1.00 Ref	
G/T	276	44	264	42	1.16 (0.88–1.52)	0.307
T/T	195	31	189	30	1.14 (0.85–1.53)	0.392
G	594	47	618	49	1.00 Ref	
T	666	53	642	51	1.07 (0.92–1.26)	0.359

^a^Crude odds ratio (OR); 95 % CI, confidence interval at 95 %

^b^Chi-square

A haplotype analysis was performed to estimate the interaction between the G135C polymorphism of *RAD51* gene, as well as between the G172T polymorphism of *RAD51* and endometrial cancer occurrence. The haplotype analysis, according to the wild-type of G135G–G172G, showed a high incidence of C135C–G172G, C135C–G172T and C135C–T172T genotypes (see Table 4). The combined G135C–G172G genotype decreased the risk of endometrial cancer occurrence (*p* < 0.05).

**Table 4:** Frequency of the *RAD51* haplotypes among endometrial cancer patients and control subjects

Haplotypes *RAD51*-135–172	Patients (*n* = 630) *N* (%)	Controls (*n* = 630) *N* (%)	OR (95 % CI)^a^	*p* ^b^
G/G–G/G	45 (7.1)	75 (11.9)	1.00 Ref.	
G/G–G/T	48 (7.6)	51 (8.1)	1.56 (0.91–2.69)	0.134
G/G–T/T	45 (7.1)	60 (9.5)	1.25 (0.73–2.13)	0.492
G/C–G/G	20 (8.1)	72 (11.7)	**0.46 (0.24–0.85)**	**0.020**
G/C–G/T	45 (7.1)	114 (11.4)	0.66 (0.39–1.09)	0.134
G/C–T/T	45 (7.1)	75 (11.9)	1.00 (0.59–1.68)	0.887
C/C–G/G	120 (14.3)	57 (9.0)	**3.50 (2.15–5.70)**	**<0.0001**
C/C–G/T	165 (26.2)	69 (10.9)	**3.98 (2.50–6.34)**	**<0.0001**
C/C–T/T	96 (15.2)	57 (9.0)	**2.81 (1.71–4.60)**	**<0.0001**

Data in boldface are statistically significant

^a^Crude odds ratio (OR); 95 % CI, confidence interval at 95 %

^b^Chi-square

Histological grading was related to *RAD51* G135C and G172T polymorphisms. Histological grades were evaluated in all the cases (*n* = 630). There were: G1, 180 cases; G2, 420 cases and G3, 30 cases. Grades 2 and 3 were accounted together for statistical analysis (see Table 5). Some correlation was observed between the *RAD51* G135C and G172T polymorphisms, and endometrial cancer invasiveness. An increase was observed, regarding 135C allele frequency (OR 1.43; 95 % CI 1.09–1.88, *p* = 0.011) and 172T allele (OR 3.81; 95 % CI 2.90–5.01, *p* < 0.0001) in G1 patients, according to FIGO classification [31]. That increase was statistically significant (*p* < 0.05). A tendency for an increased risk of EC was observed with the occurrence of T172T genotype of *RAD51* polymorphism (*p* < 0.05).

**Table 5:** Dependence of genotypes and frequencies of *RAD51* gene polymorphism alleles on tumor grade in endometrial cancer patients

Grade^a^	Endometrial cancer patients		OR (95 % CI)^b^	*p* ^c^
	G1 (*n* = 180)	G2 + G3 (*n* = 450)		
	Number (%)	Number (%)		
*RAD51* G135C				
G/G	29 (16)	100 (22)	1.00 Ref	
G/C	35 (20)	100 (22)	1.21 (0.68–2.12)	0.610
C/C	116 (64)	250 (56)	1.60 (1.00–2.55)	0.062
G	93 (26)	300 (33)	1.00 Ref	
C	267 (74)	600 (67)	**1.43 (1.09–1.88)**	**0.011**
*RAD51* G172T				
G/G	20 (11)	139 (31)	1.00 Ref	
G/T	50 (28)	226 (50)	1.53 (0.87–2.69)	0.168
T/T	110 (61)	85 (19)	**8.99 (5.20–15.55)**	**<0.0001**
G	90 (25)	504 (56)	1.00 Ref	
T	270 (75)	396 (44)	**3.81 (2.90–5.01)**	**<0.0001**

Data in boldface are statistically significant

*n* = 630

^a^According to FIGO criteria

^b^Crude odds ratio (OR); 95 % CI, confidence interval at 95 %

^c^Chi-square

We did not find any association of the *RAD51* polymorphisms in the patients group with cancer progression assessed by endometrial cancer staging (*p* > 0.05) (data not shown).

Our data did not demonstrate any statistically significant correlation between *RAD51* polymorphisms and the risk factors for endometrial cancer, such as BMI (body mass index), HRT (hormone replacement therapy), uterine bleeding, endometrial transvaginal ultrasound, diabetes and hypertension and women with endometrial cancer, here again erase the remark “(data not shown)”.

## Discussion

In the presented study, the role of polymorphisms was studied in DNA DSB repair *RAD51* gene, the polymorphisms being regarded as risk factors for endometrial cancer in a case setting. The following SNPs were considered in the homologous recombination (HRR) pathway: *RAD51* G135C and G172T.

Homologous recombinational repair plays a critical role in repairing DNA damage [4, 5, 32]. The RAD51 protein is a core component of DNA double-strand break repair by HRR [9].

The cells, which are deficient in this gene product, are defective in homologous recombination and demonstrate genomic instability [9].

The cellular reaction to DNA damaging agents can modulate the susceptibility towards tumor development [33]. This reaction is mainly determined by the efficacy of DNA repair, which may, in turn, be influenced by the variability of DNA repair genes, expressed by their polymorphisms [34–37].

The variability of *RAD51* repair gene could contribute to the protein biosynthesis level [38–40].

It is supposed that the polymorphism of the *RAD51* gene has been associated with interindividual differences in the basal steady state level of its protein [9, 10]. The *RAD51* gene has been mapped to 15q14-15 chromosome and is highly polymorphic in nature [9, 10].

As mentioned in the “Introduction” above, the involvement of *RAD51* in DNA repair determines its potential role in maintaining the genomic stability, which is disturbed in various malignancies [11–17]. Therefore, the problem of genetic variability of the *RAD51* gene for tumor development is worth to be studied.

A G to C substitution at position 135 and G to T substitution at position 172 of the *RAD51* gene (5′-untranslated region) have been described as single nucleotide polymorphisms (SNPs) [8]. Both polymorphisms are located in the regulatory element of the *RAD51* promoter and are suggested to be associated with messenger RNA stability and expression [9, 10].

The results of several previous studies suggest that RAD51 plays an important role in repair of double-strand breaks in DNA [33, 41]. Defects of genes, involved in DSB repair, often lead to better cancer development [33].

The *RAD51* polymorphisms were found to be associated with various cancer diseases [12, 17, 42, 43] but little data are available on the association or its lack in endometrial cancer.

According to several research endeavors described in the current literature, polymorphisms in the 5′ untranslated region of *RAD51* gene may contribute to endometrial carcinogenesis [28–30].

Recent reports introduce the role of G135C polymorphism in the development of endometrial cancer [28, 30].

However, the study was carried out on a relatively small patient population, thus the obtained results cannot be considered as definitive and require further, more extensive evaluations, performed on bigger groups of patients.

In view of the potentially significant role of the DNA repair machinery for more intensive cancer development, it is important to know whether the *RAD51* gene polymorphism can account for the appearance of endometrial cancer occurrence. Therefore, we analyzed the role of G135C and G172T genetic variations in the homologous recombination repair gene and for the risk of developing EC.

In the presented study, the PCR–RFLP technique was used to screen 630 endometrial cancer patients for *RAD51* polymorphisms.

A significant difference was found in the incidence of allele distribution among investigated samples. In the presented study, the incidence of 135C allele in the examined patients was higher than that of 135C allele in control samples (69 vs. 46 %, respectively). The genotype distribution in the patients differed from that, expected from the Hardy–Weinberg equilibrium, with an overrepresentation of 135C allele. Moreover, 135C/C genotype increased the risk of EC. It is possible that the presence of C allele remains in some linkage disequilibrium with another, so far unknown, mutation, located outside of the coding region in the *RAD51* gene, which may be of importance for the RAD51 concentration in plasma and more severe cancer development.

On the other hand, no significant difference was found among the G172T genotypes in tumor and normal endometrial tissues. We realize that this may have been due to the rather small population enrolled into the study or due to de novo mutations in investigated samples.

In the reported study, the G135C polymorphism of *RAD51* gene and G172T of *RAD51*, were correlated with endometrial carcinoma progression. 135C and 172T alleles were associated with an increased risk of grade 1 endometrial cancer.

In conclusion, the reported study is another evidence for the significance of G135C and G172T genotypes in EC grading.

Thus we conclude that our observations may be an important signal, prompting to appreciate the role of *RAD51* in EC development and likely triggering further studies on this interesting subject.
